# Can losartan and blood pressure control peri arteriovenous fistula creation ameliorate the early associated left ventricular hypertrophic response a randomised placebo controlled trial

**DOI:** 10.1186/1756-0500-5-260

**Published:** 2012-05-29

**Authors:** Dominica Zentner, Eugenie Pedagogos, Anthony Yapanis, Sofie Karapanagiotidis, Alison Kinghorn, Athanasia Alexiou, Geoffrey Lee, Matija Raspudic, Anuradha Aggarwal

**Affiliations:** 1 Dept of Cardiology, Royal Melbourne Hospital, Grattan St, Parkville, 3050, Australia; 2 Dept of Nephrology, Royal Melbourne Hospital, Grattan St, Parkville, 3050, Australia

**Keywords:** Arteriovenous fistula, Blood pressure, Echocardiography, Transthoracic, Hypertrophy, Left ventricular, Haemodialysis

## Abstract

**Background:**

Haemodialysis results in a left ventricular hypertrophic response. It is unclear whether tight blood pressure control or particular medications might attenuate this response. We sought to determine, in a pre-dialysis cohort on atenolol, whether Losartan might attenuate left ventricular hypertrophy post arteriovenous fistula creation in end stage kidney disease.

**Materials and methods:**

Placebo controlled double blind randomisation of 26 patients to fixed dose atenolol plus fixed dose losartan or placebo occurred 1 day prior to fistula creation. Pre-randomisation echocardiography was repeated at 1 week and 1-month. Measurement was undertaken of blood pressure, heart rate, brain natriuretic peptide, serum creatinine and estimated glomerular filtration rate. The primary pre-specified endpoint was the change in left ventricular mass at 1 month. Non-parametric statistical comparison was performed within and between groups.

**Results:**

There was no difference in left ventricular mass between our groups 1-month post fistula creation. In the entire cohort, change in left ventricular mass was driven by changes in blood pressure and volume loading. Blood pressure changes correlated with left ventricular mass changes seen shortly post arteriovenous fistula creation, suggesting blood pressure control during this time period may be an important part of the management of end stage kidney disease.

**Conclusions:**

We did not see an advantage with the use of losartan with respect to diminution of the LVM response. However, our demonstrated change in LVM was relatively small compared to previous literature and suggests a possible role for beta blockade as a neurohormonal modulator around the time of arteriovenous fistula creation.

**Trial registration:**

Clinical trials.gov (NCT00602004).

## Background

Left ventricular hypertrophy is common in the end stage kidney disease population [1] prior to dialysis commencement, where it appears to relate to both hypertension and anemia [2]. Creation of an arteriovenous fistula (AVF) for long-term renal replacement therapy further increases left ventricular wall thickness causing left ventricular hypertrophy (LVH)[3,4] and increased mass (LVM) and cardiac chamber enlargement [5,6]. Occasionally, a more extreme response occurs with the development of high output cardiac failure following creation of an AVF [7]. Reversibility of these changes in cardiac parameters has been documented post renal transplant in patients in whom functional AVF are closed [3,8,9].

Both systolic heart failure and end stage kidney disease (ESKD) have a heightened sympathetic nervous system (SNS) response [10,11] .In heart failure there is evidence of mortality benefit with ACE inhibitors and beta-blockers [12,13]. It is unknown whether these medications might attenuate the cardiac response to AVF creation.

A small number of studies have suggested that losartan, an angiotensin receptor blocker (ARB), might diminish the LVH response to volume overload. This has been seen in a rat model of volume overload with induction of aortic regurgitation [14], and in humans on established haemodialysis (HD) [15,16], although one of these papers also differed with respect to BP variability between the losartan and the control group [15]. It is accepted that effective treatment of hypertension achieves a decrease in LVM in the wider population [17] and in patients on HD [18], although whether an incremental drug class specific effect on LVH may exist once BP is well controlled is less clear.

A mild state of volume overload has been seen to occur early post AVF creation [19], with changes in both cardiac function and increases in atrial natriuretic peptide (ANP) levels despite a lack of change in left atrial (LA) size. Current echocardiography guidelines recommend measurement of LA volume (LAV), not size, as a better marker of atrial remodelling with a more significant relationship to cardiovascular disease [20]. Concurrently, a move to measurement of brain natriuretic peptide (BNP) instead of ANP has occurred in the arena of heart failure research and management [21].

BNP, a more stable measure in renal disease than NT-proBNP [22], correlates with left ventricular mass index (LVMI) in end stage kidney disease (ESKD) patients and is known to increase with volume expansion and cardiomyocyte stretch [22,23]. BNP has been shown to predict mortality in HD patients without heart failure [23], and both ventricular arrhythmias and mortality in a meta-analysis of patients with reduced ventricular ejection fraction (EF) [21].

We sought to determine, in a prospective double blind randomised trial, whether the addition of losartan to atenolol in a population undergoing AVF creation attenuated the increase in LVM. All patients were commenced on atenolol in order to minimise, as much as possible, differences either in type of antihypertensive medications or blood pressure control achieved between the losartan and placebo groups.

Additionally, we assessed BNP level and changes in cardiac output (CO), EF and LAV in response to AVF creation.

## Results

### Recruitment and power

**Figure 1:**
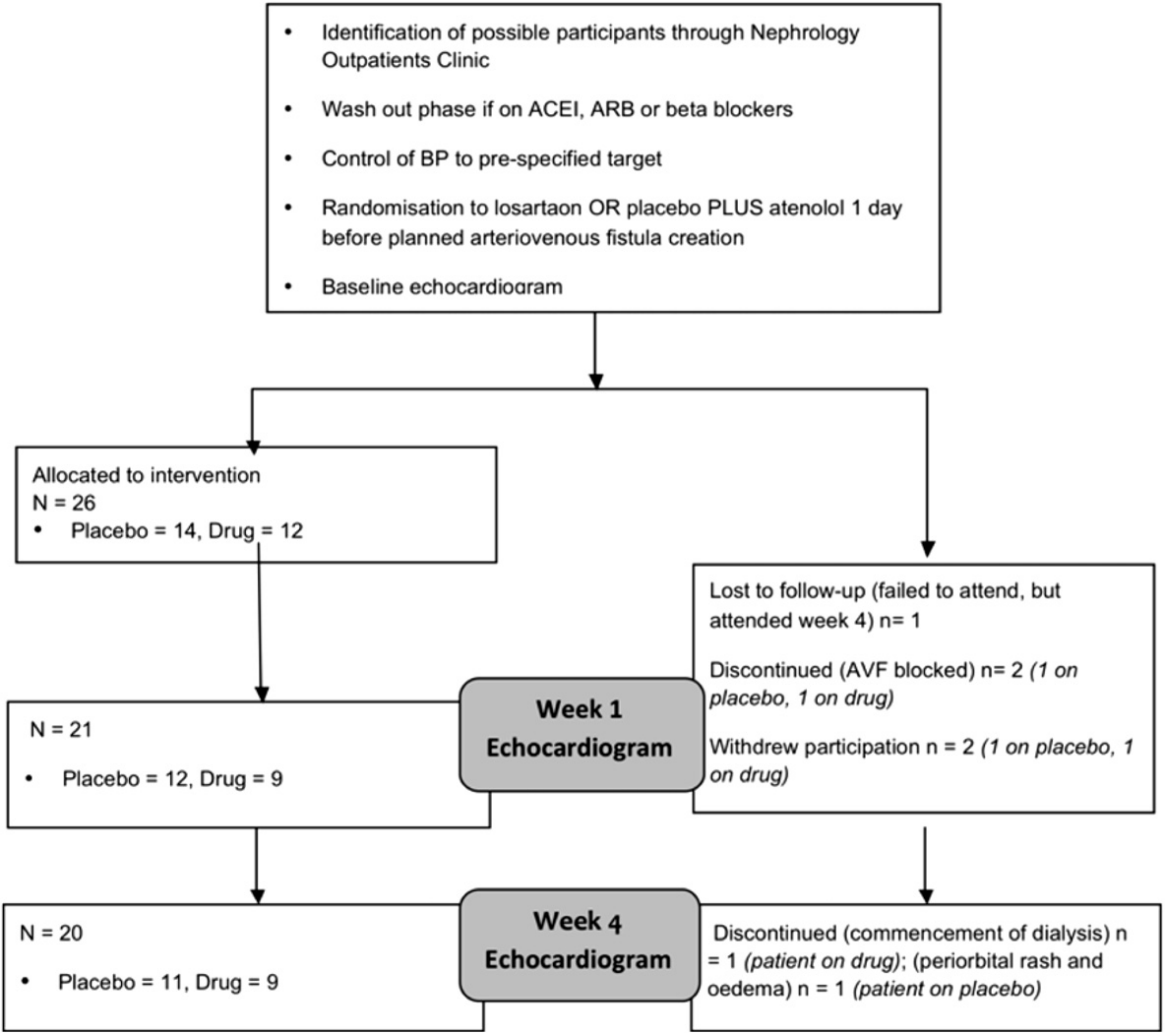
Flowchart of study methodology and participation at each time point.

Recruitment proved to be difficult, with numbers of patients being considered for an elective AVF creation as they approached clinical need for dialysis being insufficient to achieve the sample size envisaged. Recruitment was thus terminated after a 2-year period, during which a total of 26 patients were recruited. This sample size gave us 67% power to detect a difference between the losartan and the placebo group. All 26 patients had a baseline echocardiogram, 21 underwent a week 1 study and 20 underwent a week 4 study (Figure 1).

### Treatment phase

Randomisation resulted in 14 patients in the atenolol plus placebo and 12 patients in the atenolol plus losartan arm. The investigators were blinded with respect to which patients were taking either placebo or losartan.

Baseline characteristics in the groups are shown in Table 1. The Losartan group was statistically significantly older (2p = 0.019), with no significant differences seen between the groups in sitting SBP, DBP or heart rate, or in body weight or body surface area (BSA). Median BNP levels were higher with a greater IQR in the losartan group, but this was not statistically significant.

**Table 1:** Baseline characteristics at randomisation

**Variable**	**Losartan (n = 12)**	**Placebo (n = 14)**
Age (y)	66.5 (56–73)	57.5 (38.5–63)^a^
Gender (F: M)	4: 8	6: 8
eGFR	9.5 (6–16)	12 (10–14.5)
Hemoglobin	111.5 (104–128.5)	117 (111–138)
Creatinine	465 (322.5–597.3)	440 (339–480)
sSBP (mmHg)	1142 (132–160)	137 (130–153.5)
sDBP (mmHg)	74 (72–80)	78 (70–86)
sHR (bpm)	64 (60–74)	68 (61–74)
Weight (kg)	74.2 (71.4–88.3)	80 (70–89.4)
BSA (kg/m^2^)	1.90 (1.76–2.10)	1.91 (1.79–2.06)
BNP (ng/L)	285 (65–1194)	163 (63–303.3)
Additional antihypertensive medications (n) (non protocol)	2.0 (0.5–2.5)	2.0 (0–3)

This table shows the gender spread, age, estimated glomerular filtration rate (eGFR), sitting (s) systolic (SBP), diastolic blood pressure (DBP) and heart rate (HR), weight and body surface area (BSA), brain natriuretic peptide (BNP) measurement and the additional number of antihypertensive medications (excluding protocol medications) in the atenolol plus losartan (losartan) and atenolol plus placebo (placebo) groups. Data is expressed as median (interquartile range) other than for gender, which is expressed as a ratio. Statistical comparison was undertaken by the Mann–Whitney *U* test. Comparison of the gender spread was undertaken with a Chi square test. Statistically significant differences are marked by the letter a (^a^). (Please note: non protocol medications with an antihypertensive effect included: calcium channel blockers, diuretics, prazosin, glyceryl trinitrate patch and moxonidine).

### Baseline echocardiography

At baseline, echocardiography results between the placebo and losartan groups showed no significant difference between the randomised groups with respect to median LVM, RWT, CO, E/E’, LAV or EF (Table 2).

**Table 2:** Results at baseline and 1 month in both the Losartan and Placebo groups

**Variable**	**Losartan (A) baseline**	**Losartan (B) 1 month**	**Placebo (A) baseline**	**Placebo (B) 1 month**	**P**
sSBP (mmHg)	142 (132–160)	129 (117–149)^a^	137 (130–153.5)	138 (127–141.5)	A ns B ns
sDBP (mmHg)	74 (72–80)	70 (64–84)	78 (70–86)	79 (70–85.5)	A ns B ns
sHR (bpm)	64 (60–74)	62 (56–80)	68 (61–74)	72 (66–78)	A ns B ns
BNP	285 (65–1194)	325 (206–1798.5)	163 (63–303.3)	214 (144–277.5)	A ns B ns
LVM (gm)	199.1 (148.5–225.2)	218.4 (146.5–262.2)^a^	182.7 (143.5–205.0)	173.8 (151.1–204.9)	A ns B ns
LVMI (gm/m^2^)	99.6 (72.8–114.3)	83.5 (69.8–112)	91.3 (77.6–106.8)	92.7 (78.4–107.5)	A ns B ns
RWT	0.40 (0.32–0.45)	0.36 (0.32–0.41)	0.40 (0.34–0.47)	0.37 (0.35–0.42)	A ns B ns
CO (L/min)	5.3 (4.5–5.6)	5.9 (4.5–7.3)	4.4 (3.7–5.9)	5.3 (4.6–6.9)	A ns B ns
E/E’	15.0 (10.0–21.0)	12 (8.8–17.3)	11.4 (9.8–14.0)	10.4 (8.8–13.2)^a^	A ns B ns
LAV (mLs)	66.5 (54.8–75.6)	74.3 (59.8–80.5)	65.0 (50.1–82.2)	65.1 (59.4–81.3)	A ns B ns
EF (%)	55 (47–57.8)	52.5 (49.3–56.8)	52.5 (47.8–58.8)	52 (51–60)	A ns B ns

This table shows the sitting systolic (SBP) and diastolic (DBP) blood pressure and heart rate (HR), brain natriuretic peptide (BNP), left ventricular mass (LVM) and LVM index (LVMI), relative wall thickness (RWT), cardiac output (CO), E/E’, left atrial volume (LAV) and ejection fraction (EF) in the atenolol plus losartan (losartan) and atenolol plus placebo (placebo) groups at baseline (A) and 1 month (B). Data was compared utilising non-parametric tests: between groups (Mann–Whitney U) in the column labelled P (next to A for comparisons at baseline and next to B for comparisons at 1 month) and within groups (Wilcoxon), (from baseline to 1 month) when statistical significance was denoted by the letter a (^a^) if P <0.05 compared to baseline. (P denotes a two-tailed p value).

### Echocardiography at 1 week

The only statistically significant result between the groups at 1 week was in E/E’ which was greater in the losartan group (p = 0.04). There was no difference between the groups in BP, HR, BNP, LVM, LVMI, RWT, CO, LAV or EF (data not presented further). In both the losartan and placebo group however, CO and CI were statistically greater by 1 week compared with baseline (losartan: CO P = 0.015, CI P = 0.025; placebo: CO P = 0.026, CI P = 0.016). Both CO and CI had trended back down towards the baseline value by 1 month, with no statistically significant difference seen on comparison with either the baseline or 1 week result.

### Echocardiography at 1 month

At 1 month, there were no significant differences seen between the placebo and losartan groups in blood pressure, heart rate, BNP, LVM, CO, LAV, EF.

Compared with baseline, at 1 month the losartan group had lower sitting SBP (2p = 0.042) and a higher LVM (2p = 0.028).(Table 3) However, there was no change in LVMI and this is presumably due to the change in weight between these time points although this did not reach statistical significance [median 74.2 (71.4–88.3) kg vs median 80.8 (74–90.4), 2p = 0.07]. The losartan group also had a statistically significant decrease in hemoglobin during this time, from 111.5 (104–128.5) to 106 (101.5–117.0) (2p = 0.013). This may have affected the absolute LVM increase, as a strong negative correlation was present at baseline between hemoglobin and LVM (r = −0.7, 2p = 0.018). At 1 month, the correlation between hemoglobin and LVM was no longer statistically significant, possibly because of lower numbers of results being available. The possibility of volume overload/overhydration exists to explain this acute change in weight and haemoglobin. Of interest however, the BP decrease during this period (median −17/- 4 mmHg) appears to suggest that significant volume overload was unlikely*.*

In contrast, at 1 month there were no differences in clinical or biochemical data compared with baseline in the placebo group. In the echocardiographic data, the only difference in the placebo group was E/E’, which decreased from 11.4 (9.8–14) to 10.4 (8.8–13.2) (2p = 0.036). No significant change in hemoglobin or weight was seen.

### The hemodynamic effects of creating an AVF

In view of both the smaller sample size than anticipated and the fact that BP did not differ between the groups at the time points of interest, we decided to investigate the hemodynamic changes documented in the entire cohort, regardless of whether they had received losartan or placebo. All subjects in this study had a clinically patent AVF on the day of each echocardiogram.

### Percentage changes in hemodynamics and BNP

When considered as one group (Losartan and placebo combined) the cohort showed significant early hemodynamic changes in response to the creation of an AVF. By 1 week, compared with baseline, EF, CO and LAV had risen a median of 8% (p =0.05), 24% (p = 0.001) and 19% (p = 0.031) respectively. Sitting SBP decreased 5% (ns) and LVMI decreased by 1.5% (ns). The biochemical endpoints were not significantly different.

In contrast, the 1-month results, compared to the 1-week data, demonstrated no further statistically significant changes. It is possible that the smaller subject numbers for comparison between 1 week and 1 month precluded the finding of a statistically significant difference.

### Correlations

Baseline LVM and haemoglobin were significantly negatively correlated (r = − 0.5, 2P = 0.008) for LVM and (r = −0.4, 2P = 0.032) for LVMI.

### Linear regression

Univariate linear regression demonstrated that the statistically significant variables with 1 month LVM were baseline LVM (p < 0.001), baseline hemoglobin (2p = 0.003) and baseline BNP (2p = 0.025). Neither SBP nor medication arm was statistically significant. Multivariate linear regression with these variables resulted in only baseline BNP (2p < 0.001) and baseline LVM (2p < 0.001) remaining statistically significant.

Multivariate analysis of the prercentage change in LVM (change in LVM/original LVM) during treatment demonstrated that the percentage change in sitting SBP (2p = 0.003) and in BNP (2p = 0.016) were both significant.

## Discussion

To our knowledge, this is the first study of prophylactic neurohormonal modulation in the early setting of AVF creation. The timing of our data collection (1 week and 1 month post AVF creation) was predicated on the studies of Iwashima [7] and Ori [19], both of which documented changes in CO and LV size within a fortnight of AVF creation. They did not calculate LVM but reported a statistically significant increase in end diastolic dimension without a significant change in wall thickness. Although we doubled the follow-up time compared to these studies, and recruited more participants than either, it is possible that the early endpoint at 1 month precluded documentation of the maximum change in LVM. Recently published data however supports the timing of our study with respect to haemodynamic measures: 21 subjects studied at 2 weeks and 3 months post AVF creation showed little subsequent alteration in blood pressure or CO beyond the first assessment timepoint [24]. Unfortunately, that study provides no information on LVM,which was our major endpoint.

Increased LVM is an important risk factor for cardiovascular morbidity and mortality, both in the wider population [25] and HD patients.[26] In the general population, treatment of hypertension decreases LVH and LVM [27] as well as the clinical end points of cardiovascular morbidity and mortality [28].

We did not find that losartan attenuated the LVM response to AVF creation, with 67% power in terms of numbers recruited suggesting that the negative finding is likely to be a true finding. All our patients were treated to the same BP goals, with addition or up-titration of antihypertensive agents as required. This allowed us to also look at the entire cohort with respect to the acute and short-term cardiac effects of AVF creation. In doing so, the importance of BP on LVM is evident. Within a month of AVF creation, our cohort showed a change in LVM in response to changes in systolic blood pressure and volume loading (as evidenced by the relationship with BNP). This is an important finding, as the role of BP control in ESRD is debated, particularly with respect to whether the same threshold for and goals of treatment should apply as in the wider population [29]. Data in the non-ESRD population suggests benefits beyond BP lowering with some antihypertensive agents, particularly renin-angiotensin system antagonists [30]. There is little data on the response to different antihypertensive medications in the ESRD population.

Some significant differences also exist between our study and the published literature addressing the use of ARBs. The study from which we determined our power calculation had a very significant diminution in LVM over a 6-month period in 10 patients on losartan [16]. Notably, a greater change was also seen in hemoglobin (average difference of 2.2 g/dL compared with 1.8 g/dL in the ACEI and 0.7 g/dL in the calcium channel blocker arm). No treatment target for BP was identified, though the article states that no differences existed between the groups. From the information provided it appears that none of these patients were prescribed a BBl. Two other studies have a greater number of patients and longer follow up, but were also performed after commencement of HD, not during fistula creation. Mitsuhashi [15] recruited 40 patients on established HD, of whom half were treated with losartan. A positive effect was seen on LVM after 6 and 12 months, evident despite an apparently matched decrease in BP between the losartan and control groups. Concomitant BBl was prescribed in a small number of patients (2 in the control group and 3 in the losartan group). BP at recruitment was lower in our study than in either of these losartan positive studies. One other study, of patients 2 months post commencement of HD, was similar in size to ours (24 patients in total, divided into 2 arms) of which one arm received a high dose of losartan (100 mg) thrice weekly [31]. They found regression in LVM over 12 months in the losartan treated group vs placebo. There was a significant decrease in SBP in this group compared with pre treatment values that appears greater than the change seen in the control group on the graphical representation provided.

Data with respect to the use of beta-blockers in the HD population, in the absence of systolic heart failure, is limited [32]. Pathophysiologically, a rationale for their prescription exists given the recognition that HD is a state of chronic SNS activation [10,33,34]. The failure to see a difference between the two groups in our study may be because all patients were also on a beta-blocker, decreasing the additional effect that could be ascribed to short-term use of losartan. It is possible that the lack of significant change in BNP during this study supports a beneficial effect of beta-blockade, as this has previously been documented to increase significantly post AVF creation [7]. A retrospective study of long-term dialysis patients has previously shown a lower risk of heart failure and death in patients who were on a beta-blocker compared to those without [35]. A prospective study of dialysis patients with systolic heart failure showed an improvement in functional status and EF with a diminution in LV volume over 12 months with use of carvedilol [36].

### Limitations

This is a small study that did not recruit the necessary numbers for a definitive conclusion as to whether losartan diminishes the LVM response to AVF creation in patients where BP was treated to target and in whom beta-blockers were ubiquitously prescribed.

## Conclusions

Our aim was to determine whether pre-emptive use of an ARB decreased the LVM response to AVF creation. We were not able to demonstrate a difference between our groups with respect to LVM change, but of interest the difference in LVM in our entire cohort was not significant over this period of time. Likewise, SBP did not alter significantly. However, when the change in these variables was considered as a percentage change from baseline in the entire cohort, they were significantly correlated. We conclude that control of BP during the peri AVF creation time may be of long-term benefit and suggest that beta blockade may provide complementary benefit in altering the cardiac remodelling effects of fistula creation. Our study suggests that a future study with prophylactic beta blocker use in ESRD prior to AVF creation may be warranted.

## Methods

### Recruitment phase

All patients with ESKD (GFR = 15–30 mLs/min) at the Royal Melbourne Hospital (a tertiary referral hospital) being considered for elective AVF creation in preparation for haemodialysis were offered participation in the study.

### Inclusion and exclusion criteria

Patients had to be > 18 and < 85 years of age. Pre-menopausal women had to be on contraception in view of potential randomisation to losartan with its known teratogenic effects [37]. Baseline echocardiography was required to demonstrate normal EF (defined as > 45%).

Active exclusion criteria were hyperkalaemia, recent (within 6 months) myocardial infarction or stroke, uncontrolled hypertension (SBP > 160 mmHg or DBP > 100 mmHg), atrial fibrillation, greater than mild aortic stenosis or greater than moderate mitral regurgitation and any patient factor that would interfere with protocol compliance.

### Screening and randomisation phase

After screening for suitability, a 2-week wash out period was undertaken for all patients on angiotensin converting enzyme inhibitor (ACEI), angiotensin receptor blocker (ARB) or beta-blocker (BBl). Other antihypertensive agents were introduced if clinically required during this time. Patients were randomised after 4 weeks of adequate BP control (SBP range of 130–150 mmHg, DBP range of 80–90 mmHg) into either atenolol (25 mg) *and* placebo or atenolol (25 mg) *and* losartan (50 mg) arms in a 1: 1 ratio the day prior to AVF creation. Of the 26 patients, 25 had a native AVF (13 proximal, 12 distal) and 1 had a graft. Haemodialysis was only commenced in 1 patient during the study and this patient’s participation was terminated at that time (Figure 1).

### Treatment phase

Patients were clinically assessed at 1 and 4 weeks post AVF creation by a brief history and examination, with recording of sitting systolic (SBP) and diastolic (DBP) blood pressures and heart rate (average of 3 measures over 5 minutes). Blood was drawn for biochemical assessment.

Echocardiography, with a pre specified protocol, was performed at these time points on a Vivid 7 (GE Medical) digital machine, by 1 of 3 cardiac technologists. Studies were stored and subsequently reported by 1 of 2 cardiologists blinded both to patient identity and treatment arm.

LVM was calculated as LVM (g) = 0.8[1.04(LVIDD + IVST + PWT)^3^ – LVIDD^3^ +0.6 where LVIDD = left ventricular internal dimension in diastole, IVST = interventricular septal thickness and PWT = posterior wall thickness [20]; biplane LV volume determination allowed calculation of biplane Simpson’s EF; diastolic function was determined by assessment of mitral valve inflow (E and A wave velocity) and with pulse wave tissue Doppler imaging (TDI) assessment of septal E’ and subsequent calculation of preload as E/E’. CO [38] and LAV [20] were calculated and right atrial pressure [11,20] and right ventricular systolic pressure (RVSP) [38] were estimated according to established guidelines. Determination of relative wall thickness (defined as RWT = {2 × posterior wall thickness}/left ventricular internal diameter in diastole}) allowed categorisation into concentric and eccentric hypertrophy according to RWT ≥ 42 and RWT < 42 respectively [39].

### End point

The pre specified primary endpoint was a between group difference in the change in LVM from baseline to 1 month.

### Ethics approval

The RMH Human Research and Ethics Committee granted ethics approval (HREC 2006.059) and all participants gave informed consent. The study adhered to the principles of the Declaration of Helsinki. Janssen Cilag provided funding for losartan and matching placebo tablets. The company had no input into the research design or results analysis.

### Statistics

Power calculation (90% at α = 0.05) determined sample size to be 24 patients per arm based on a published finding of a 15% LVM difference in a study comparing losartan with both enalapril and amlodipine group [16].

Data is presented as median (interquartile range). Non-parametric tests were undertaken for comparison between and within groups. Percentage changes from baseline to 1 week and from 1 week to 1 month are presented. Spearmans rho test was used for correlation. Univariate and multivariate linear regression was undertaken to explore the primary end point of LVM at 1 month. All statistics were explored using SPSS (PASW 18.0).

## Authors’ contributions

DZ – reported 2/3’s of the echocardiograms; statistical analysis; wrote the manuscript (all revisions and final manuscript). EP – study conception; patient recruitment; patient assessment; critical revision of paper; reviewed final manuscript. AY – reported 1/3 of the echocardiograms; reviewed final manuscript. SK – performed echocardiograms; reviewed final manuscript. AK - performed echocardiograms; reviewed final manuscript. AA- performed echocardiograms; reviewed final manuscript. GL – study conception; ethics submission; reviewed final manuscript. MR – assisted with patient recruitment; collected blood samples and liaised with the laboratories involved; oversaw randomisation and dispensing of medication; reviewed final manuscript. AA – study conception; assisted with ethics submission; critical revision of paper; reviewed final manuscript. All authors read and approved the final manuscript.

## Competing interest

Funding provided by Jansen Cilag for purchase of losartan and matching placebo tablets.
